# Effect of a butyrate-fortified milk replacer on gastrointestinal microbiota and products of fermentation in artificially reared dairy calves at weaning

**DOI:** 10.1038/s41598-018-33122-6

**Published:** 2018-10-08

**Authors:** Eóin O’Hara, Alan Kelly, Matthew S. McCabe, David A. Kenny, Le Luo Guan, Sinéad M. Waters

**Affiliations:** 1 Teagasc Animal & Bioscience Research Department, Teagasc Grange, Dunsany, Co Meath Ireland; 2 UCD School of Agricultural and Food Science, University College Dublin, Belfield, Co, Dublin, Ireland; 3 Faculty of Agricultural, Food and Nutritional Sciences, University of Alberta, Edmonton, Alberta Canada

## Abstract

Enrichment of calf diets with exogenous butyrate has shown promise as a promotor of calf growth and intestinal development. However, the impact of dietary derived butyrate on the gut microbiota and their potential role, in turn, as mediators of its effect on calf growth and development is not known. Here, the effects of butyrate supplementation on rumen and hindgut microbiota and fermentation profiles were assessed in 16 Holstein-Friesian bull calves randomly assigned to one of two groups: Control (CON) fed conventional milk replacer or Sodium-Butyrate (SB – added to milk replacer) from days 7 to 56 of life. In the colon, total short chain fatty acid (SCFA), propionate and acetate concentrations were increased by SB (P < 0.05). 16S rRNA gene amplicon sequencing showed cecal abundance of butyrate producers *Butyrivibrio* and *Shuttleworthia* were decreased by SB (P < 0.05), while that of the propionate producer *Phascolarctobacterium* was higher (P < 0.05). *Mogibacterium* is associated with impaired gut health and was reduced in the cecum of SB calves (P < 0.05). These data show that the beneficial effects of SB on growth and performance occur in tandem with changes in the abundance of important SCFA producing and health-associated bacteria in the hindgut in milk-fed calves.

## Introduction

The digestive physiology of calves changes dramatically in the first weeks and months of life, and the transition from a nominal monogastric to functional ruminant is fraught with challenges^1,2^. The occurrence of gastrointestinal disorders in this period is a source of substantial economic loss in dairy production systems, responsible for around 10% of calf mortality^3^. With rising concerns surrounding the prophylactic and growth-promoting use of antibiotics in livestock production promotion^4^, there is much interest in the development of synthetic and natural alternatives to promote bovine intestinal health and development in early life.

The gastrointestinal tract (GIT) microbiota of ruminants and other production animals is well established as a key feature underscoring animal health, development and productivity^5^. In adult cattle, the rumen microbiota is the predominant feed-degrading microbial community. However, up to 20% of milk solids may pass to the hindgut for digestion during the milk feeding phase, placing elevated importance on the hindgut microbiota in this period^6^. Short chain fatty acids (SCFAs) are organic acids produced throughout the intestinal tract by microbial fermentation, and are vital in the stimulation of intestinal growth and development^7,8^. The antimicrobial properties of SCFAs and their natural presence in the mammalian digestive tract suggested that SCFA-derived feed additives may be an alternative to conventional antimicrobials in livestock production^9^. Among the most prominent of the luminal SCFAs, butyrate has been investigated for its effectiveness in enhancing animal growth and intestinal integrity and development in young livestock, with promising results^10,11^. Butyrate is the primary energy source for rumen epithelial cells and colonocytes, which are important mediators of water, mineral, and nutrient absorption^12^. Butyrate inclusion in both milk replacer and solid feed has been shown to have beneficial effects on both intestinal development and animal growth in young livestock^9,10,13,14^.

Enteric disorders in calves are associated with microbial dysbiosis in the gut^15^, and thus the health-promoting effects of exogenous butyrate may be underpinned by modulation of the GIT microbiota. There is evidence that encapsulated butyrate can reduce enteric pathogen colonisation in swine and poultry^13,16,17^, and direct infusion of butyrate into the mature cow rumen caused significant changes to the resident microbiota^18^. However, there are little data concerning the effect of long-term supplementation of butyrate on GIT microbial communities in pre-weaned calves. Given the established impact of butyrate on animal growth and intestinal development, we hypothesised that provision of a butyrate-fortified milk replacer impacts microbial communities throughout the GIT while improving host performance. Therefore, the objectives of this study were to assess microbial composition and fermentation in the rumen and hindgut at weaning in dairy calves offered milk replacer enriched with butyrate.

## Materials and Methods

### Ethical statement

All procedures involving animals were approved by University College Dublin, animal research ethics committee (UCD AREC), under licence from the Irish Department of Health and Children in accordance with the Cruelty to Animals Act (Ireland 1897) and European Community Directive 86/609/EC.

### Animal study

Forty-four male Holstein-Friesian calves (13 ± 5 days of age) were obtained from one dairy farm, and were housed on one research farm for use in this study. Calves were blocked according to age and body weight and were randomly assigned to one of two treatment groups; CON (fed unaltered milk replacer, n = 22) or SB (encapsulated sodium butyrate included in milk replacer at 4 g/kg of DM daily, n = 22). Calves were placed on a standard 56-day calf rearing program upon arrival at the research farm, with milk replacer (12.5% solids; Crude Protein 23% and Crude Fat 20%; Blossom^TM^, Volac, UK) offered at 6 L/day via an automatic feeder (Forester Tecknik, KFA3-MA3). Concentrates (rolled barley 26.5%; soya bean meal 25%; maize 15%; beet pulp 12.5%; soya hulls 12.5%; molasses 5%; minerals & vitamins 2.5%; vegetable oil 1%; Nutriad, Belgium) and water were offered on an *ad libitum* basis throughout the experimental period. Detailed dietary composition is presented in Supplementary Table S1. All calves were in good health throughout the experimental period. Calves were weaned over a 7-day period (D49–56) via gradual reduction in the allocation of milk replacer. On D56, eight animals from each group were randomly selected for euthanasia using an intravenous overdose of pentobarbital sodium (Dolethal^TM^, Vetoquinol UK, 1.4 ml/kg live body-weight). Death was confirmed by lack of an ocular response and heartbeat. Digesta samples from the rumen, cecum, and colon were collected, immediately snap frozen on liquid nitrogen, and stored at −80 °C pending molecular analysis. A further digesta sample was collected from both the rumen and colon (representative of the total hindgut SCFA profile, as previously shown^19^) for SCFA analysis. These samples were passed through four layers of cheesecloth and stored in H_2_SO_4_ at −80 °C prior to SCFA analysis using gas chromatography.

### Microbial DNA extraction

Frozen digesta from the cecum, colon and rumen was ground under liquid nitrogen to a fine powder. Total DNA was extracted using the RBB + C method as previously described^20^; approximately 250 mg of ground frozen sample was subjected to repeated bead beating followed by column purification with a QIAGEN DNeasy Stool Kit (Qiagen, UK). DNA quantity and purity were assessed by two consecutive readings at A260 and A280nm on a Nanodrop 1000 spectrophotometer, and visualisation with UV light in a 0.8% agarose gel. Samples with DNA purity values <1.6 were re-extracted, as were samples of low concentration (<100 ng/µl). Mean quantity and purity values in each gastrointestinal region are provided in Supplementary Data S5.

### Microbial profiling using amplicon sequencing

Amplicons of the V4 hyper-variable region of the 16S rRNA gene were prepared using Illumina Nextera chemistry, as previously reported^21^. DNA concentrations recorded on the Nanodrop were used to normalise each sample to a concentration of 100 ng/µl with molecular water. A 25 µl PCR reaction using 20 ng of DNA, and KAPA Hi-Fi PCR (New England Biolabs Inc.) mix was prepared using 515 F/806 R primers^22^ to simultaneously characterise bacterial and archaeal members using the following cycle programme: 95 °C for 3 minutes, and 25 cycles of: 95 °C for 30 sec, 55 °C for 30 sec, 72 °C for 30 sec, with a final elongation step of 72 °C for 30 seconds. Amplicons were purified using the QIAGEN QIAquick PCR Purification Kit. A second PCR step was performed to add Illumina dual indices and Nextera^TM^ adapters to the purified fragments (Illumina, San Diego, CA, USA). Following another column purification, the barcoded amplicon products were combined into two pools in equimolar quantities to ensure adequate sequencing coverage. Each pool was subjected to gel (QIAquick Gel Extraction Kit, Qiagen) and column purification (QIAquick Purification Kit, Qiagen) to remove primer dimers and any residual agarose. Purified pools were quantified by qPCR using the KAPA SYBR FAST Universal kit with Illumina Primer Premix (New England Biolabs Inc.). Pools were then diluted and denatured according to the Illumina MiSeq library preparation guide. A 6 pM amplicon library was spiked with 30% denatured and diluted PhiX Illumina control library (version 3, 12.5 pM), and subjected to sequencing on the Illumina MiSeq platform with one pool per run.

### Sequence data quality control and pre-processing

Demultiplexed paired end reads were trimmed and filtered to remove low quality reads and bases (Phred quality score threshold of 20), and simultaneously merged together using the BBTools suite^23^. The resulting merged reads were then size selected to retain only reads ± 2 standard deviations from the mean read length, to minimise spurious OTU creation. Finally, merged pairs were combined into a single file for downstream processing using the Quantitative Insights Into Molecular Ecology (QIIME v.1.9) tool^24^.

### Bioinformatic Analysis

Operational taxonomic unit (OTU) identification using a similarity level of 97% was carried out using the open reference picking method implemented in QIIME^24^. A representative sequence from each identified OTU was then aligned against the reference Greengenes database (v.13_8)^25^. A graphical representation of the phylogenetic trees created in QIIME was made using the Interactive Tree of Life software^26^. The raw and unfiltered OTU table created in QIIME was imported into R to create a Phyloseq class object^27^. α-diversity was computed by first randomly subsampling (rarefying) the OTU table to the lowest read number, to reduce bias due to differential sequencing depth. The Shannon and Chao1 metrics were used to assess diversity and evenness of the rumen and hindgut microbiota. β-diversity was calculated in a similar manner, with a Bray Curtis Dissimilarity matrix constructed from the rarefied OTU table. A cluster dendrogram using Ward linkage equilibrium was generated from the same OTU table in R. Principle Coordinate Analysis (PCoA) was performed in Phyloseq and used to visualise these distance matrices in 2-dimensional space. OTUs represented by <2 sequences were filtered and removed. Relative abundances of taxa at the phylum, family, and genus levels were computed in R.

### Statistical Analysis

Permutation based Analysis of Variance (PERMANOVA) analysis based on the Bray Curtis Dissimilarity Matrix was carried out in R using the Vegan package to compare microbial structure between groups and GIT region^28,29^. Taxonomic abundances at the phylum and genus levels were compared across treatments (within GIT compartment) using a Wald parametric test, offered within the DESeq2 Bioconductor package in R^30^. A false-discovery rate (FDR) threshold of 0.15 was used to determine statistical significance^31^. Only taxa present at ≥0.01% of all 16S rRNA sequences in either treatment group were considered present. Exploratory investigation of taxonomic profiles revealed two outlier animals (one from each group), and they were removed from subsequent analysis leaving a total of 7 animals in each treatment group.

## Results

### Animal performance

This experiment was conducted in association with a larger study designed to examine the effect of SB supplementation on the performance, feed efficiency and immune status of artificially reared dairy calves. Briefly, from this perspective, calves supplemented with SB tended (P = 0.08) to have a higher pre-weaning growth rate compared to CON (0.69 versus 0.59 kg/day). At weaning SB calves (80.2 kg) were 3.1 kg heavier than the CON group (76.9 kg) with bodyweight difference detected from day 42 to weaning (P = 0.08). Bodyweight differences between treatments were not evident prior to this (P > 0.1). Total DMI was not different between dietary treatments but pre-weaning SB supplementation tended (P = 0.08) to improve feed efficiency (measured using feed conversion ratio) of the calves (SB; 1.7:1 compared to CON; 2.5:1; P = 0.07). Feed intakes and growth rates are presented in supplementary data.

### Fermentation profiles in the rumen and hindgut at weaning

Short chain fatty acid profiles of the rumen and colon contents at weaning are presented in Table 1. Colonic concentrations of total SCFA, propionate, and acetate were higher for SB fed calves (P < 0.05). SB supplementation reduced ruminal butyrate concentration (P < 0.05), but total SCFA concentration was unaffected.

**Table 1:** The effect of SB inclusion in milk replacer on Short Chain Fatty Acid (SCFA) profiles in the rumen and colon.

SCFA Profiles						
	Rumen			Colon		
Item	CON	SB	P-value	CON	SB	P-value
**Total SCFA Concentrations (mmol/L)**						
Acetate	90.56 ± 7.51^a^	78.46 ± 1.93	*NS* ^b^	39.48 ± 3.49	60.84 ± 6.03	0.01
Propionate	62.43 ± 7.51	58.26 ± 1.73	*NS*	10.77 ± 1.20	17.06 ± 2.00	0.02
Butyrate	16.21 ± 1.17	11.49 ± 0.57	0.04	3.56 ± 0.40	5.01 ± 0.84	*NS*
Isobutyrate	0.71 ± 0.37	0.33 ± 0.06	*NS*	0.53 ± 0.05	0.50 ± 0.09	*NS*
Valerate	4.91 ± 0.61	3.43 ± 0.09	*NS*	0.75 ± 0.13	0.85 ± 0.09	*NS*
Isovalerate	1.79 ± 0.28	1.05 ± 0.08	*NS*	0.37 ± 0.06	0.31 ± 0.06	*NS*
Total VFA	176.44 ± 16.02	152.82 ± 3.67	*NS*	55.46 ± 4.87	84.57 ± 8.60	0.02
**Molar Proportions of SCFA**						
Acetate	0.517 ± 0.01	0.513 ± 0.003	*NS*	0.712 ± 0.01	0.721 ± 0.01	*NS*
Propionate	0.346 ± 0.01	0.379 ± 0.004	*NS*	0.192 ± 0.01	0.200 ± 0.01	*NS*
Butyrate	0.094 ± 0.004	0.077 ± 0.004	*NS*	0.064 ± 0.003	0.057 ± 0.01	*NS*
Isobutyrate	0.003 ± 0.002	0.001 ± 0.001	*NS*	0.010 ± 0.001	0.007 ± 0.001	*NS*
Valerate	0.028 ± 0.003	0.023 ± 0.001	*NS*	0.014 ± 0.001	0.010 ± 0.001	*NS*
Isovalerate	0.011 ± 0.002	0.007 ± 0.001	*NS*	0.007 ± 0.001	0.004 ± 0.001	*NS*

P-values were obtained using a Monte-Carlo permutational t-test in R.

^a^Mean ± SEM. ^b^Not significant.

### Microbial structure and diversity in the rumen and hindgut in response to SB

Amplicon sequencing of rumen and hindgut digesta samples from calves at weaning yielded a total of 10,348,464 high quality reads, with an average of 215,593 ± 75,380 sequences per sample. Taxon abundance was agglomerated at the genus and phylum levels for comparisons across treatments, and relative abundances of all detected taxa are summarised in Supplementary Data S2.

**Figure 1:**
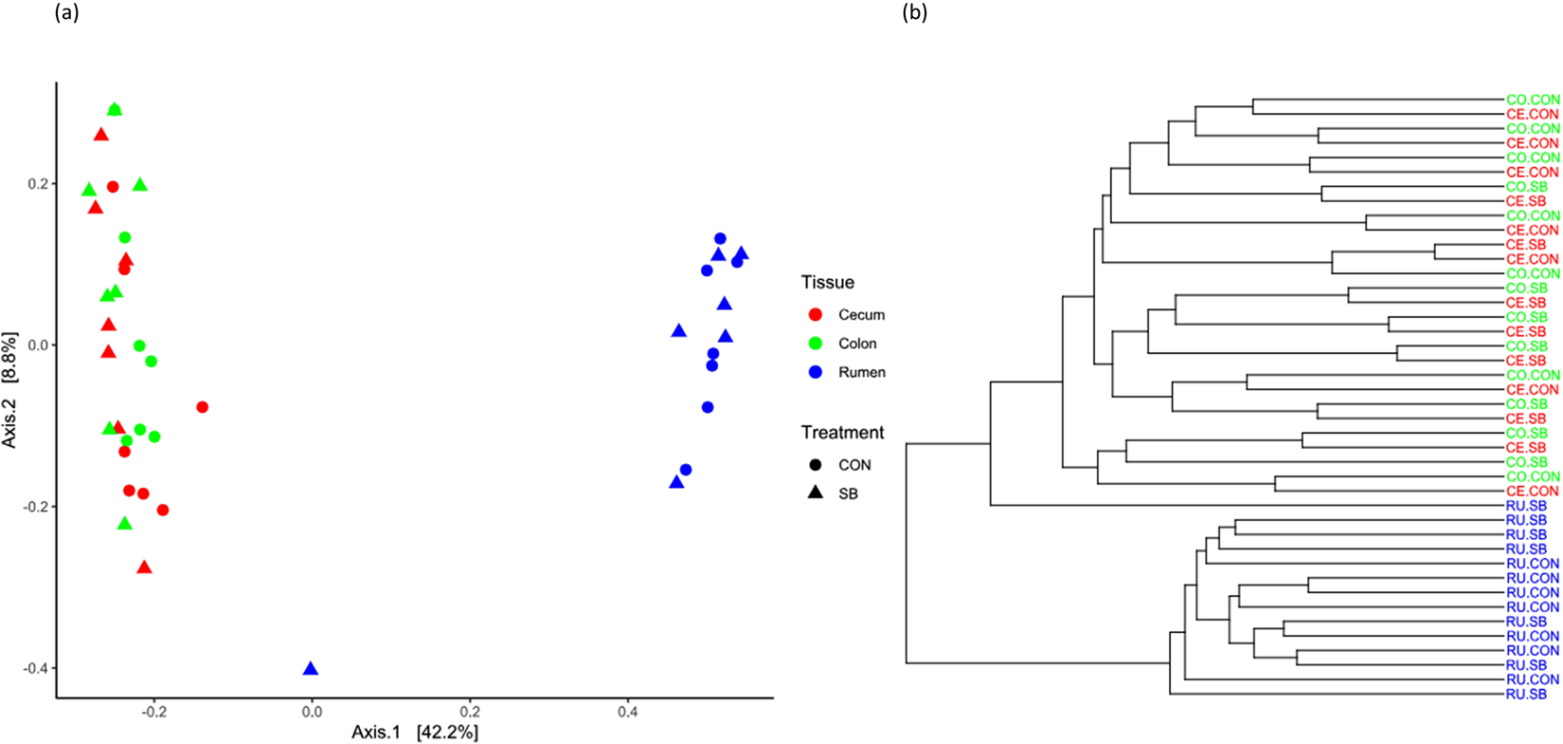
(**a**) Principle Coordinate Analysis plot and (**b**) cluster dendrogram generated based on the Bray-Curtis dissimilarity matrix of operational taxonomic units (OTUs) present in the rumen, cecum, and colon.

Alpha diversity measured using the *Shannon* index was not affected by treatment in any region studied, though was higher in both hindgut regions than in the rumen (P < 0.05; Table 2). The *Chao1* index of species richness was lower in the rumen of SB animals (P < 0.05), but was similar across treatments in the hindgut (Table 2), and was higher in the colon than both other compartments (P < 0.05; Table 2). Principle coordinate anlaysis (PCoA) and cluster analysis showed some evidence of separation according to treatment, independent of GIT region in the hindgut (Fig. 1a,b), but comparisons using PERMANOVA failed to detect any differences (P > 0.05; Table 3**)**. There was, however, clear separation according to gastrointestinal region, with the rumen community clustering away from both hindgut regions (P < 0.05), while both hindgut regions appeared to harbour a similar microbial community (Fig. 1a,b).

**Table 2:** Comparisons of alpha diversity metrics in the rumen, cecum, and colon of calves at weaning.

α-Diversity								
	Chao1				Shannon			
	Overall	CON	SB	P-value	Overall	CON	SB	P-value
Rumen	1698.0^a^	1887.2	1508.7	0.01	3.6^a^	3.7	3.6	0.15
Cecum	1728.7^a^	1630.3	1827.0	0.30	4.9^b^	4.8	5.0	0.28
Colon	2849.0^b^	2827.3	2870.7	0.87	5.1^b^	5.1	5.1	0.76

Significant differences according to gastrointestinal region are denoted with different letters.

**Table 3:** Comparing microbial communities between treatments and gastrointestinal region in the rumen, cecum and colon.

β-Diversity					
Treatment			GIT Region	F-value	P-value
	F-value	P-value			
Rumen	1.04	0.37	Rumen vs. Cecum	21.44	0.001
Cecum	1.30	0.16	Rumen vs. Colon	21.15	0.001
Colon	1.28	0.12	Cecum vs. Colon	0.82	0.700

P-values obtained using PERMANOVA analysis based on Bray Curtis similarity matrices.

### Microbial composition in the rumen and hindgut in response to SB

#### Rumen

**Figure 2:**
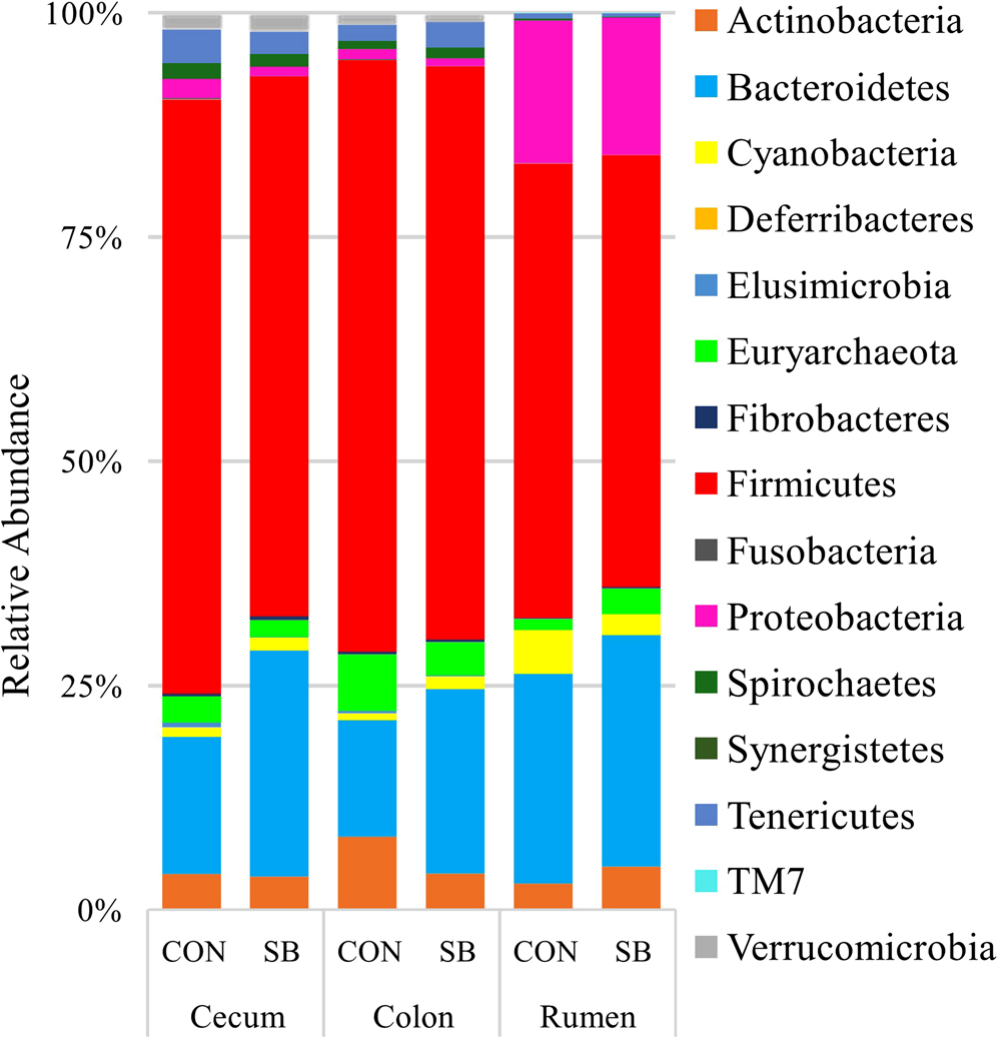
Stacked bar chart of microbial abundances at the phylum level, calculated as a percentage of total 16S rRNA reads within each group.

**Figure 3:**
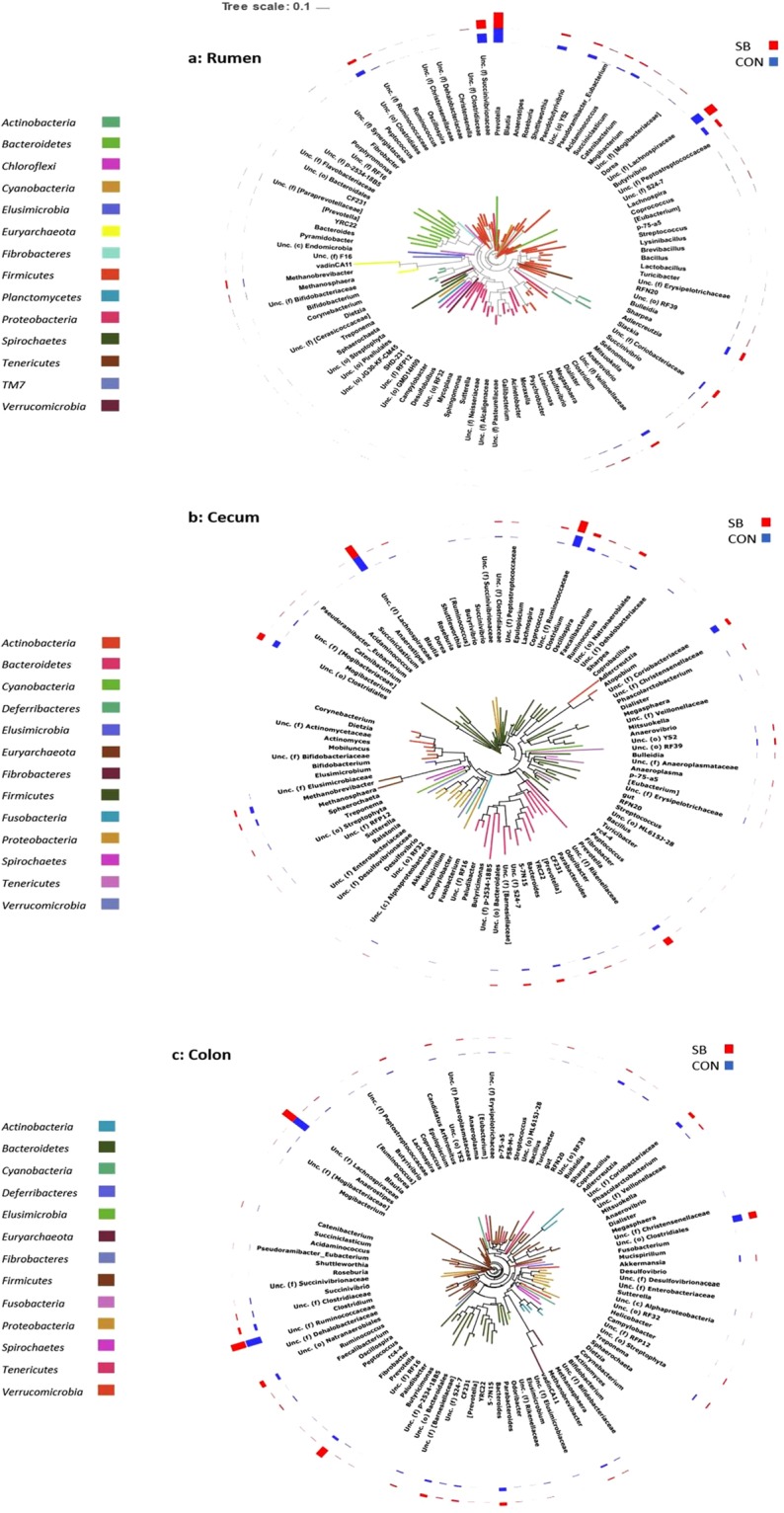
Phylogenetic Trees of (**a**) rumen, (**b**) cecum, and (**c**) colon microbiota. A phylogenetic tree file was built from a multiple sequence alignment generated in QIIME. OTUs were agglomerated at the genus level in R. The trees were visualised using the Interactive Tree of Life (ITOL) software package.

Among the bacterial phyla detected in the rumen, *Bacteroidetes*, *Firmicutes*, and *Proteobacteria* were predominant, followed by *Actinobacteria* and *Cyanobacteria* (Fig. 2), while the remaining minor phyla (<1% 16S rRNA reads) are presented in Fig. 2. Archaea were represented by the *Euryarchaetota* phylum. 60 genus-level assignements were reported from the rumen, with *Prevotella*, *f.Succivibrionaceae* and *f.Lachnospiraceae* predominant at weaning, regardless of dietary treatment. Notably, only 48.14% of reads recovered from the rumen could not be confidently assigned at the genus level. This is reflected in the high abundances of f.*Succinivibrionaceae* (f = family level, unassigned at genus level in QIIME), f.*Lachnospiraceae* and o.*Clostridiales* in the rumen samples, as well as a further 21 unclassifed genus-level taxa (Supplementary data S2, Fig. 3a). Comparisons of taxon abundance in DESeq2 between SB and CON animals showed no statistically significant effect of dietary treatment on the rumen microbiota at either phylum or genus level following adjustment into FDR.

#### Hindgut

Twelve bacterial phyla and a single archaeal phyla were detected in the hindgut, among which *Defferibacteres* was unique to the colon **(**Fig. 2**)**. Like the rumen, a significant proportion of 16S rRNA reads recovered from the cecum and colon could not be resolved taxonomically to the genus level (~59%). 93 and 88 genera were detected in the cecum and colon, respectively. Genera annotated only as f.*Lachnospiraceae* and f.*Ruminococcaceae* were the most abundant in both compartments (Fig. 3b,c**)**. There was a minor impact of treatment on composition of the hindgut microbiota. For instance, in the colon, *Prevotella* was enriched in SB animals (P < 0.05). In the cecum, several taxa were different between treatments; as in the colon *Prevotella* (4.31–9.48%) was numerically higher in the SB cohort, but this difference was not signficant, possibly due to the large inter-animal variaiton observed (Supplementary Data S2).

An additional 9 genera were different between dietary treatments in the cecum (P < 0.05); *Shuttleworthia* (0.01 vs. 0.06%), *Butyrivibrio* (0.13 vs. 0.81), *Sharpea* (0.32 vs. 1.09%), and *Mogibacterium* (0.12 vs. 0.26%) were all reduced by SB supplementation (P < 0.05), as well an unidentified member of the f.[*Mogibacteriaceae*] (0.65 vs. 1.56%) (Fig. 3b**)**. A genus belonging to the Cyanobacterial *YS2* order was increased by SB, as were *Lachnospira* (0.13 vs. 0.06%), *Phascolarctobacterium* (1.40% vs. 0.66%), and a genus annotated as *p-75-af* belonging to *Erysipelotrichaceae* (0.31 vs. 0.11%; P < 0.05). A single genus from the *Tenericutes* phylum classified only as o. *ML615J-28* was also increased in the SB group (0.19 vs. 0.04%; P < 0.05). Additionally, an undetermined genus assigned to the *Coriobacteriaceae* family was reduced by SB in the cecum (3.96 vs. 8.17%; P < 0.05; Fig. 3b).

## Discussion

The beneficial effects of dietary butyrate supplementation (often included in salt form as calcium or sodium butyrate) on animal growth and intestinal development have been demonstrated in calves^10,14,32^, chickens^17^ and pigs^33^. While there is now an established body of evidence supporting the potential of butyrate as a beneficial feed additive, its impact on the gut microbiota is unknown. In adult animals, hindgut fermentation typically provides 5–10% of dietary energy, but this may be elevated during the pre-weaning phase of calf growth, when up to 20% of ingested milk solids may pass to the hindgut^6,34^. Thus microbial fermentation in the cecum and colon is an important host energy source during this period^6^. Given that the importance of the hindgut in feed digestion is accentuated during early ruminal development, it is of interest to ascertain what changes may occur in the microbiota and fermentation patterns following SB supplementation. In a previous study, our group showed positive effects on growth and efficiency when dairy calves were supplemented with SB^35^. Here, we provide evidence that such improved performance is accompanied by changes in microbial composition and fermentation in the hindgut compartments, while the rumen microbiota is mostly unaffected.

### SB does not induce substantial changes in the rumen microbiota or fermentation profile

In terms of bacterial composition, the rumen microbiota was unaffected by SB. However, species richness (assessed using the *Chao1* estimator) was lower in the SB animals, indicative of a greater number of sparsely abundant OTUs being present the rumen of CON animals than the SB group. Interestingly, we also observed a reduction in ruminal butyrate concentration in the SB cohort. The digestive physiology of the milk-fed calf effectively precludes entry of liquid feed into the reticulorumen via action of the reticular groove^36^, and so these changes are likely due to an indirect effect of SB on the rumen microbiota, as the exogenous butyrate in the milk replacer did not enter the rumen. Such indirect influences of SB on the rumen have previously been observed; SB-fortified MR significantly improved rumen growth and papillae development compared to calves fed conventional MR^37^, but we did not observe such effects in the present study where rumen papillae length, width, and perimeter were not affected by SB supplementation (data not shown). Thus, though we observed a reduction in the concentration of ruminal butyrate, this does not appear to have had any detrimental effects on rumen development. It is possible that if the excess dietary butyrate was absorbed in the gut, it may have reduced the requirement for ruminal butyrate in the SB calves. It is also worth noting that many inconsistent results have been reported in the literature when butyrate or its derivatives are used as supplements in livestock diets, as recently reviewed^12^. Nonetheless, this suggests cross-talk mechanisms may exist between the lower gut and the rumen and warrant further investigation. In studies where SB was included in calf starter, significant development of the rumen epithelium was observed^10,14^, and future work should also examine changes in the rumen microbiota and fermentation profiles when calves are supplemented with SB in solid feed.

### Sodium butyrate modifies the hindgut microbiota and fermentation profiles in early life

The microbial profiles of the cecum and colon were highly similar. No significant clustering was observed in the PCoA plot according to treatment within either compartment, but finer shifts in the microbial profile were evident in both. In the colon, the proportion of *Prevotella* was increased by SB, with a similar numerical increase observed in the cecum. Enrichment of *Prevotella* in the colon and stomach of neonatal piglets has previously been reported following SB supplementation^13^. *Prevotella* is established as a primary member of the mammalian gut ecosystem, comprising species capable of fermenting a wide range of non-cellulosic plant polysaccharides and protein^38^. *Prevotella* spp. positively correlated with intestinal butyrate concentrations in growing pigs^39^, and it is possible that excess dietary butyrate reaching the colon conferred a competitive advantage on *Prevotella*, as they are not notable butyrate producers^40^, aligning with the significant reduction in known butyrate producing taxa discussed below. Supplementing the diet of neonatal piglets and poultry with SB has previously been reported to reduce the abundance of known gut pathogens (e.g *E. coli*)^41^. We did not observe similar effects in our study, which may be attributable to differences in analytical approach (e.g. qPCR for specific scour causing bacteria). We did detect *Escherichia* in our dataset, but its proportion was very low (<0.005% of total 16S rRNA sequences) and so was not considered in our final analysis. This highlights a limitation of amplicon sequencing surveys, whereby potentially important taxa may be under- or over-represented due to variation in 16S rRNA gene copy number among microbial species^42^.

We observed most evidence of microbial manipulation through SB supplementation, in the cecum. Most notably, the abundances of several important SCFA producers were changed. *Phascolarctobacterium* rapidly converts succinate to propionate in the gut^43,44^. The higher abundance of this genus in the cecum of SB animals may have contributed to improved growth via increased host energy substrate, as propionate is the primary precursor for gluconeogenesis in ruminants^44^. This, combined with our observation of higher levels of propionate and total SCFA, provides evidence that improved rates of bacterial fermentation in the hindgut may also contribute to SB-driven performance improvements, as well as the increased activation of the IGF-1 pathway previously reported^9^. Abundances of known butyrate-producing *Butyrivibrio* and *Shuttleworthia* were reduced in the cecum under SB supplementation, suggesting that exogenous butyrate suppresses microbial biosynthesis of butyrate in the gut. The reduction of the lactate producer *Sharpea* may also contribute to lower microbial butyrate as lactate is an intermediate molecule formed by bacterial action in the GIT. Lactate is usually rapidly utilised for SCFA (primarily butyrate) synthesis, as accumulation can lead to harmful acidotic conditions^45,46^. While the mechanisms and occurrence of ruminal acidosis has been extensively investigated in cattle^47–49^, there is little knowledge of the prevalence of hindgut acidosis in calves. Lactate was not measured in the present study, but our results suggest that lactate metabolism may be an important intermediary in the response of the gut microbiota to exogenous butyrate, warranting further investigation.

Sodium butyrate supplementation in reduced cecal abundance of taxa associated with lowered gut health and integrity, and elevated inflammation. For instance, *Mogibacterium*, a known genus of the oral microbiota, was reduced in response to SB supplementation. Whilst the role of *Mogibacterium* in the gut is not fully understood, previous studies have observed a decreased fecal abundance of this genus in response to beneficial prebiotic supplementation in neonatal piglets^50^, and mucosal abundance of *Mogibacterium* was higher in the distal gut of human colorectal cancer patients than healthy controls^51^. Thus while the dearth of knowledge concerning the characteristics of *Mogibacterium spp*. in the gut ecosystem make it difficult to speculate as to why SB may affect it, it’s reduction may be indicative of favourable changes in the gut microbiota of calves fed SB. Similarly, the abundance of *Actinobacteria* was also significantly lower in the cecum of SB calves, driven by a significant reduction in a genus classified only as part of the *Coriobacteraceae* family (reported as “f__*Coriobacteraceae*;g.__” in QIIME). There were several other low-abundance genera assigned to *Coriobacteraceae* (<0.01%), so this is likely an undescribed genus or genera which may have an important role in the maintenance of gut health. Several novel members of this family have been described recently^52,53^, and further advances in our knowledge of the role of *Coriobacteraceae* in the gut may resolve the possible role of as-yet undefined *Coriobacteraceae* species in SB-driven growth improvements. The *Coriobacteraceae* in the gut have been associated with a suppression in host inflammatory response. Reduced abundance of this family was previously observed in tandem with lower detection of the pro-inflammatory IL-6 in blood plasma^54^, and so our results may indicate reduced immunogenicity among the cecal microbiota of SB fed calves.

The higher abundance of *Cyanobacteria* observed in the cecum of SB animals was driven by significant increases of a genus assigned to the *YS2* order. This highlights a wider issue concerning 16S rRNA gene investigations of intestinal microbial communities. Although *Cyanobacteria* have been widely reported as minor contributors to GIT microbial diversity in mammals^55–57^, the validity of their role in the anaerobic gut ecosystem is questionable, as many species of this phylum are native to marine environments and are notable performers of complex oxygenic photosynthesis^58^. Recent studies have revealed that the *Cyanobacteria* found in the gut are genetically dissimilar to their photosynthetic relatives, and likely diverged prior to the latter developing the capability for photosynthesis^59,60^. Two such novel *Cyanobacteria*-like lineages have been described in the human GIT to date, the *Melainabacteria*^59^, and the *Sericytochromatia*^60^, but there is not yet a consensus on the correct nomenclature^61^. Neither is it known if these novel taxa are also the same *Cyanobacteria*-derivatives present in the ruminant gut, and this warrants urgent investigation. Regardless, increased abundance of *Cyanobacteria* has not been previously reported in the gut of SB supplemented calves, suggesting a potential role of the newly described *Cyanobacteria* groups in the developing intestine, but further work is needed to confirm their role in the ruminant gut ecosystem.

### The rumen and hindgut harbour significantly different microbial communities at weaning

While patterns of microbial colonisation in the pre-functioning rumen have been the subject of several investigations recently^62–65^, there are noticeably fewer published reports concerning the hindgut microbiota of young ruminants. In agreement with the available literature, we found that the rumen and hindgut microbiota differed significantly at weaning^55,66^. In addition to lower rumen bacterial diversity, SCFA levels were higher in the rumen than in the colon, suggesting that at weaning, the rumen microbiota ferments plant biomass at a greater rate than that of the hindgut. It is likely that the greater range of secondary fermentation products entering the lower gut is the driver of the increased bacterial diversity of the cecum and colon. The bacterial profile of the rumen was similar to that previously reported in young animals and was dominated by *Firmicutes* and *Bacteroidetes*. *Bacteroidetes* have previously been reported as the predominant bacterial phyla in the rumen and hindgut of 3-week old and weaned diary calves^64,66^, and in the rumen of 6-week old lambs^67^. *Prevotella* was the most abundant bacterial genus in the rumen at weaning which is in agreement with published reports^66^. Our data showed the principal bacterial phylum *Firmicutes* was dominated by unclassified *Succinivibrionaceae* in the rumen, but that the hindgut regions harboured higher relative abundances of unclassified genera from the *Lachnospiraceae* and *Ruminococcaceae* families while the *Succinivibrionaceae* members were minor contributors. *Succinivibrionaceae* has been reported as a member of the core active rumen microbiota in adult cattle^68^, and is implicated in reduced methane formation in both ruminants and macropods^21,69,70^. The predominance of *Prevotella* and *Succinivibrionaceae* has been previously documented in the rumen of adult dairy cows^71^, but the high abundance of uncharacterised *Succinivibrionaceae* in the rumen at weaning has not, to our knowledge, been reported to date. However, caution should be exercised when comparing results of multiple amplicon sequencing surveys, as amplification primer choice can significantly bias results^72^. Popova, *et al*.^73^ and Zhou, *et al*.^7^ have previously described the hindgut methanogen populations in lambs and dairy calves, and our findings are largely similar to theirs, with *Methanobrevibacter* as the predominant genus.

Unclassified genera of the *Lachnospiraceae* were previously reported as comprising just 5.58% of faecal 16S rRNA sequences 5 days after weaning, in contrast to our observation of high abundance in the cecum and colon^66^. The same study revealed high abundance of an unclassified *Ruminococcaceae* genus in the faeces of dairy calves shortly after weaning which is consistent with our results^66^. Both taxa have been widely reported as important members of the gut microbiota, containing prominent plant polysaccharide hydrolysing species^74^. Interestingly, visualisation of the phylogenetic tree generated in QIIME shows *Prevotella* sequences recovered from the rumen appeared to cluster away from the other *Bacteroidetes* taxa (Fig. 3a), suggesting that at weaning the rumen may contain a phylogenetically distinct cohort of *Prevotella spp*. compared to that of the hindgut, where *Prevotella* sequences clustered broadly as expected (Fig. 3b,c). This warrants further investigation, given the ubiquitous and abundant presence of *Prevotella* in the mammalian digestive tract. Also evident in our dataset is the dominance of undescribed microorganisms in the mammalian GIT. Indeed, among the ten most abundant genus level taxa reported in the hindgut regions, only four (*Prevotella, Clostridium, Bacteroides* and *Ruminococcus*) were annotated as a known bacterial genus. This underlines the large number of as-yet uncharacterised bacteria that exist within the mammalian gut, and highlights the inherent difficulties in accurate compositional and functional profiling of the GIT microbiota.

## Conclusions

The data presented here and in our companion study^35^ provide evidence that the improved performance recorded for SB supplemented calves may be mediated through minor changes in the rumen and hindgut microbiota, with a particularly notable response to SB evident in the cecum. However, it is impossible to conclude whether changes in microbial composition are actively contributing to this improved growth and performance, or whether the host phenotype is driving changes in the microbial community. It is possible that the major effects of exogenous butyrate supplementation on the GIT microbiota may occur during the first weeks of life and are not evident at weaning, and indeed previous work has suggested that for maximum impact, butyrate should be supplemented from the first day of life^12^. The present study may also be limited by the fact that the calves had already undergone a weaning process (between days 49–56) when the samples were collected, and the amount of exogenous butyrate entering the GIT was thus reduced in the week preceding slaughter. It may be advantageous to collect digesta samples throughout the milk-feeding period in future studies, to assess if SB supplementation may facilitate a smoother weaning transition. Nonetheless, considering the significant differences that were still evident one week following the onset of the weaning process, SB supplementation appears to impart persistent changes on gut microbial composition and fermentation in dairy calves, and may be a candidate additive for “microbial programming” of gut microbial communities in early life^75^. In summary, we conclude that positive trends in growth rate and feed efficiency associated with SB supplementation in early life occur in tandem with changes in bacterial composition and fermentation in the hindgut. More thorough investigations using metagenomic or metatranscriptomic approaches may offer further information as to the mechanisms by which sodium butyrate modulates the gut microbial community in young ruminants.

## Electronic supplementary material

Dataset 1

Supplementary Information S1-S5
