# Supplementary material for: Effect of a butyrate-fortified milk replacer on gastrointestinal microbiota and products of fermentation in artificially reared dairy calves at weaning
Source: Sci Rep. 2018 Oct 8;8:14901. doi: 10.1038/s41598-018-33122-6 (PMC6175921; doi:10.1038/s41598-018-33122-6)
Supplement: Supplementary file 2 — Supplementary Information S1-S5 [file 41598_2018_33122_MOESM2_ESM.pdf]

Effect of a butyrate-fortified milk replacer on gastrointestinal microbiota and products of fermentation in artificially reared dairy calves at weaning.

O'Hara, E.[1,3]; Kelly. A[2]; McCabe, M. S.[1]; Kenny, D. A.,[1,2]; Guan, L.L[3]; Waters, S. M.[1*]

[1] Teagasc Animal & Bioscience Research Department, Teagasc Grange, Dunsany, Co Meath, Ireland.
[2] UCD School of Agricultural and Food Science, University College Dublin, Belfield, Co. Dublin, Ireland.
[3] Faculty of Agricultural, Food and Nutritional Sciences, University of Alberta, Edmonton, Alberta, Canada.

*Correspondence to sinead.waters@teagasc.ie

Supplementary Information

Supplementary Information S1: Nutritional composition and chemical analysis of solid feed offered.

Table S1 | Solid Diet composition

Variable	Concentration
Diet composition	
Rolled Barley, %	26.5
Soya bean meal, %	25
Maize, %	15
Beet pulp, %	12.5
Soya hulls, %	12.5
Molasses, %	5
Mineral and vitamins, %	2.5[1]
Vegetable oil, %	1
Chemical composition	
ADF, g/kg	103.1 (±6.76)
Crude ash, g/kg	68.8 (±0.91)
CP, g/kg	167.9 (±1.86)
DM, %	88.9 (±0.66)
NDF, g/kg	204.3 (±18.2)
Oil B[2], g/kg	30.8 (±0.72)

Supplementary Information S2: Growth rates of calves from birth to weaning

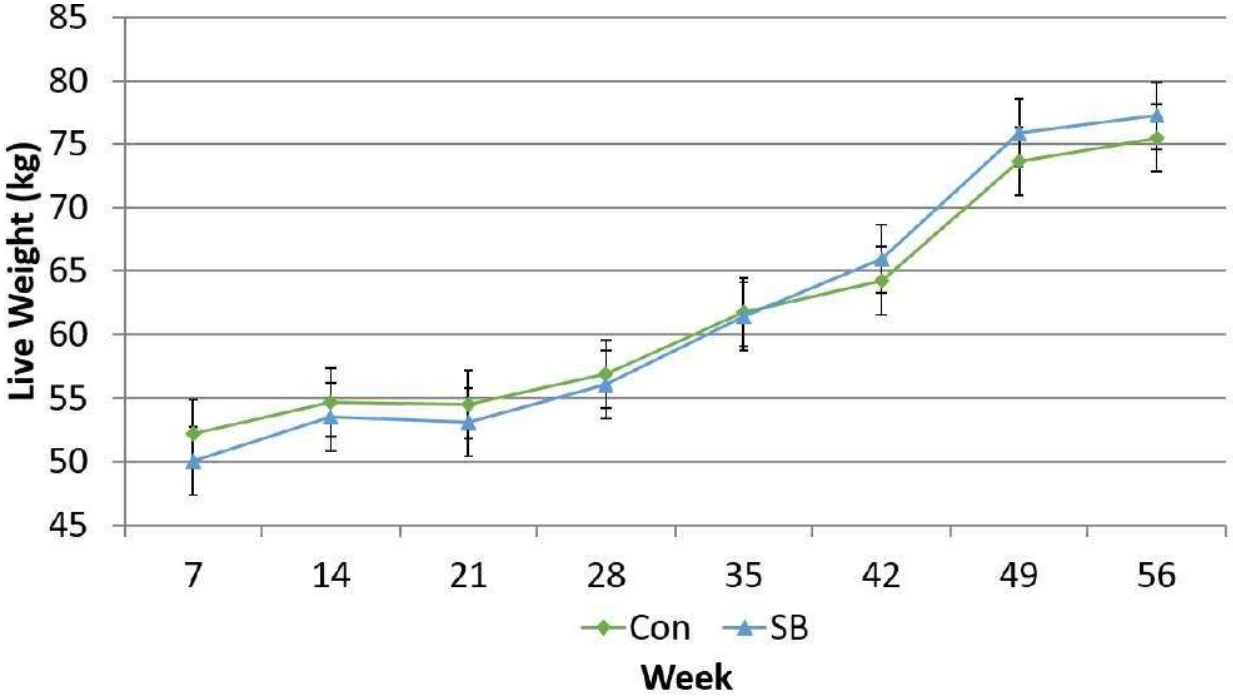

Supplementary Information S3: Solid feed consumption of calves fed MR+/- SBin the preweaning phase

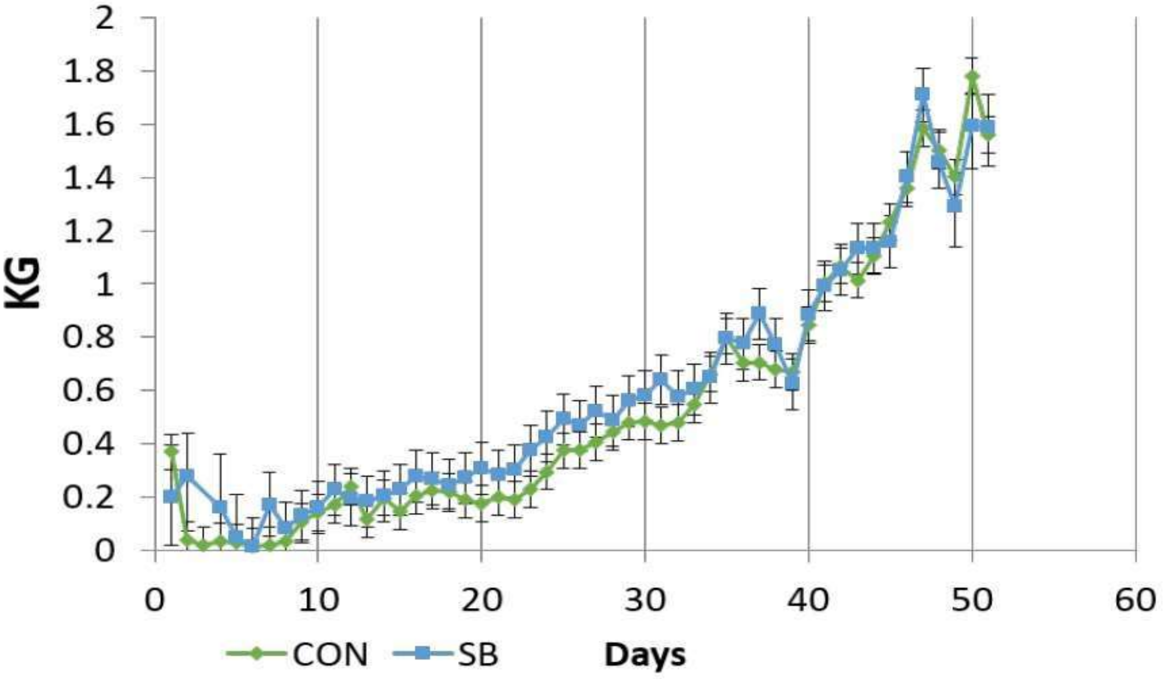

Supplementary Information S4: Milk replacer consumption of calves fed MR +/- SB in the preweaning phase

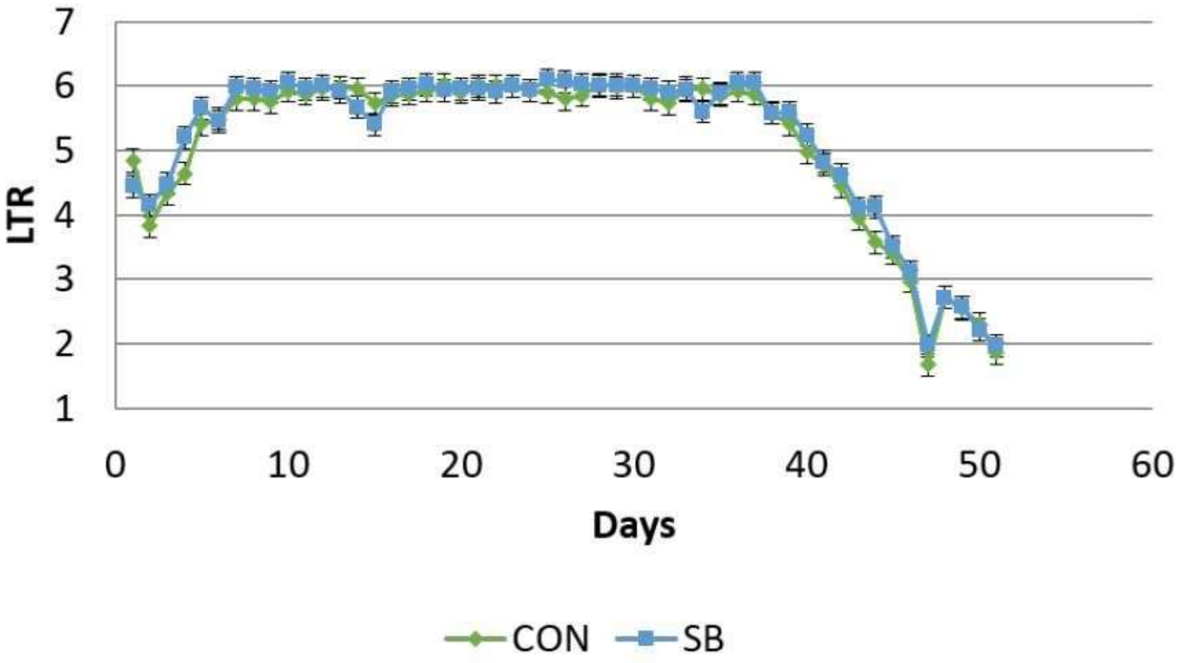

Supplementary Information S5: Details of DNA quantities and quality measurements post-extraction.

	Rumen			Cecum			Colon		
**Value**	ng/ul	260/280	260/230	ng/ul	260/280	260/230	ng/ul	260/280	260/230
**Mean**	542.4	1.8	1.9	216.2	1.9	1.8	320.0	1.9	1.8
**STDev.**	273.0	0.0	0.2	42.9	0.0	0.1	99.4	0.0	0.2
